# Four sources of pleasure associated with the experience of groove

**DOI:** 10.3389/fpsyg.2026.1881467

**Published:** 2026-09-01

**Authors:** Deniz Duman, Suvi Saarikallio

**Affiliations:** 1 Centre of Excellence in Music, Mind, Body and Brain, University of Jyväskylä, Jyväskylä, Finland; 2 Department of Music, Art and Culture Studies, University of Jyväskylä, Jyväskylä, Finland

**Keywords:** dance, groove, immersion, pleasure, social connection

## Abstract

Human behaviour is largely driven by the desire to maximize pleasure. Music, in both perception and production, is a uniquely powerful source of pleasure, engaging cognitive, bodily, psychological, and social processes. This paper focuses on groove, an everyday, effortless form of sensorimotor musical experience. While previous studies have solely examined groove as pleasurable urge to move, this paper develops a theoretical framework for understanding groove as a multidimensional source of musical pleasure. Extending beyond prior two-dimensional accounts, the framework incorporates immersive and social aspects of the experience. Gathering research findings across disciplines, four sources of pleasure are identified, that are distinguished by the primary level at which pleasure is experienced and expressed, operating at the cognitive, psychological, behavioral, and social levels: (1) pleasure arising from processing of the music, especially linked to the balance of prediction and surprise in musical structure; (2) immersive pleasure associated with a flow-like, altered attentional experience; (3) movement pleasure generated through (desire for) bodily synchronization and sensorimotor reward; and (4) pleasure rooted in social connection, synchrony, bonding, and collective participation. These interconnected levels are supported by neurochemical mechanisms that modulate attention, arousal, timing, and reward, anchoring the pleasures of groove in embodied brain–body dynamics and reflecting its biopsychosocial nature. This framework offers a more nuanced account of why groove is such a powerful, rewarding and everyday human experience. Beyond its theoretical contribution, the current work informs applied domains by highlighting various sources of pleasure that provide internal motivation, offering insights for designing music-based interventions in therapy, education, and well-being, and suggesting directions for future empirical testing.

## Introduction

“*Happiness is the meaning and the purpose of life, the whole aim and end of human existence*.” Aristotle.

The pursuit of *pleasure*^1^ has been argued to be the primary goal in life since the time of ancient Greek philosophers. Pleasure manifests in various forms, including physical sensations, emotional states, and cognitive experiences. Hence, it is a central topic for researchers across fields like psychology, anthropology, neuroscience, and behavioral sciences. From an evolutionary standpoint, the experience of pleasure serves several adaptive purposes, helping individuals navigate their environment and reinforcing behaviours that support survival and reproduction (Schultz, 2015). In psychology, *hedonism theory* posits that human behaviour is largely driven by the desire to avoid pain and maximize pleasure (Bentham, 1879). Complementing this, the *drive reduction theory* suggests that pleasure results from reducing biological or psychological needs, thereby reducing tension or arousal; in other words, satisfying drives (like eating when hungry or drinking when thirsty) leads to pleasure by restoring homeostasis; body’s state of internal balance (Hull, 1945). According to *self-determination theory* (Deci and Ryan, 2012), pleasure is associated with activities that fulfil basic psychological needs: autonomy, competence, and relatedness. This theory views pleasure as an intrinsic motivation that contributes to well-being when core needs are met. Abraham Maslow’s *hierarchy of needs theory* (Maslow, 1943) offers a similar framework for understanding pleasure as fulfilling physiological, safety, social, esteem, and self-actualization needs. According to Maslow, pleasure arises as people satisfy needs across this spectrum, contributing to a holistic sense of well-being. Similarly, Lionel Tiger’s *four pleasure model* (Tiger, 2017) categorizes pleasure types from an anthropological perspective: (1) socio-pleasure from social belonging, (2) psycho-pleasure from cognitive satisfaction and discovery, (3) ideo-pleasure from cultural, ideological, and aesthetic ideals, and (4) physio-pleasure from sensory experiences like taste, sight, and touch. As it is noted, the experience of pleasure is complex, related with survival, reproduction, satisfaction of social, physiological and psychological needs. This complexity reflects the inherently multidimensional nature of human beings.

Cognitions, emotions and behaviours (including overall well-being) are shaped by biological, psychological, and social factors (Engel, 1977). Biologically, pleasure is supported by reward processes in the brain involving dopamine and related neurotransmitters, which underpin components such as liking, wanting, and learning (Berridge and Kringelbach, 2008). Psychologically, pleasure is linked to affect regulation, motivation, and meaning making, while socially, it reinforces bonding, cooperation, and cultural practices. Reward mechanisms operate across all these levels, motivating behaviour, reinforcing learning, and ensuring survival. Accordingly, experiences that engage reward processes biologically, psychologically, and socially tend to be particularly powerful, promoting individual well-being as well as social cohesion. Musical pleasure is one such multidimensional, rewarding phenomenon. In this article, we first review the main literature on musical pleasure and groove. Building on this, we propose a framework that identifies four, potentially distinct, yet interconnected types of musical pleasure associated with the experience of *groove* (a sensorimotor form of musical experience), each shaped by specific biopsychosocial dynamics.

## Groove as a form of musical pleasure

Music, in both perception and production, is a particularly pleasurable experience, acting as a strong and rewarding stimulant that induces pleasure in various ways and elicits changes in physiological and reward-related brain networks (Blood and Zatorre, 2001; Koelsch, 2014; Krumhansl, 1997; Ferreri et al., 2019) which activates various types of pleasure ranging from sensory to social (Saarikallio et al., 2019). Music processing may primarily code immersive pleasure states, while simultaneously engaging movement-related circuits that directly induce pleasure by involving the body. One essential concept, through which musical pleasure has been studied, is *groove*, a sensorimotor form of musical engagement that reflects the desire to move to music and is closely intertwined with the pleasurable responses it evokes (Madison, 2006; Janata et al., 2012). Groove is an everyday experience. From infancy onward, people move to music easily and effortlessly (Zentner and Eerola, 2010; Kragness et al., 2023). This might be because of its rewarding nature. While it has been shown that there are multiple facets of musical pleasure, despite its potential, concept of groove has been treated as a single, undifferentiated source of pleasure. Expanding on, by gathering evidence from various disciplines, we will argue how there might be multiple sources of pleasure underlying a groove experience.

People commonly report dancing for reasons such as pleasure, entertainment, and enhancing mood and energy, among other factors (Duman et al., 2022). Empirical findings support this connection: studies have shown that the urge to move correlates strongly with reported pleasure during music listening (r = 0.82, Janata et al., 2012; r = 0.96, Witek et al., 2014; r = 0.69, Duman, 2023). While groove has often been studied as two dimensional, encompassing pleasure and urge to move (e.g., Witek et al., 2014; Senn et al., 2020), a more nuanced and updated definition has recently been brought into the literature. This multidimensional account incorporates immersive and social aspects of the groove experience: “Groove is a participatory experience (related to immersion, movement, positive affect, and social connection) resulting from subtle interaction of specific music- (such as time- and pitch-related features), performance-, and/or individual-related factors.” (Duman et al., 2024, p. 111).

This multidimensional perspective to groove also aligns with the perspective that musical pleasure in general emerges from various sources, including emotional responses, cognitive engagement, bodily involvement and social bonding. For instance, to capture the multifaceted nature of musical reward, Mas-Herrero et al. (2013) developed the Barcelona Music Reward Questionnaire (BMRQ), which identifies five distinct components of musical reward sensitivity: musical seeking (the active pursuit of music-related experiences), emotion evocation (the ability of music to induce emotional responses), mood regulation (using music to manage affective states), sensory-motor responses (the tendency of music to elicit bodily movements and chills), and social reward (music’s role in promoting social connections). More recently, a sixth dimension, *absorption*, has been introduced to the framework, reflecting the extent to which individuals experience deep immersion, attentional focus, and loss of self-awareness while listening to music (Cardona et al., 2022). Inclusion of immersion to the conceptual framing of groove experience further aligns with the concept of flow (Csikszentmihalyi and Csikzentmihaly, 1990) as well as Maslow’s notions of music as a resource for peak emotional experiences, related to intense happiness and sense of fulfilment (Lowis, 2002; Maslow, 1971). However, a review comparing pleasure drawn from music versus visual art showed that musical pleasure is actively studied in relation to emotional and physiological reactions in contrast to focus on cognitive and conceptual processes in visual art, which may be reflective of specific importance of affective-embodied aspects for musical pleasure (Tiihonen et al., 2017).

Previously, literature on groove has identified various factors shaping the experience of groove, including (1) *experiences* such as movement or wanting to move (Janata et al., 2012; Ross et al., 2016; Stupacher et al., 2013), pleasure (Janata et al., 2012; Kawase and Eguchi, 2010; Madison, 2006; Matthews et al., 2020; Witek et al., 2014), immersion or flow (Câmara and Danielsen, 2018; Duman et al., 2024; Janata et al., 2012; Stupacher, 2019) and catchiness (Bechtold et al., 2023); (2) *listener characteristics* such as musical taste and familiarity (Senn et al., 2021, 2023), musical expertise (Senn et al., 2016; Witek et al., 2017), personality traits (Duman, 2023), cultural (Etani et al., 2018; Witek et al., 2020) and developmental (Kragness et al., 2023) details; (3) *musical properties* such as certain musical styles (Bechtold et al., 2024; Frühauf et al., 2013; Senn et al., 2021), technical language (Hosken, 2020), tempo (Etani et al., 2018; Jerjen et al., 2024), frequency ranges (Hove et al., 2020; Stupacher et al., 2016), pulse and meter (Fitch, 2016), event density (Madison et al., 2011; Senn et al., 2018), rhythmic and harmonic complexity (Matthews et al., 2020; Senn et al., 2024), syncopation (Witek et al., 2014, 2017), timbre (Bechtold et al., 2024) and microtiming (Butterfield, 2010; Frühauf et al., 2013; Keil, 1995; Senn et al., 2016); (4) *listening environment* (contextual details) such as the influence of social environment (Bechtold et al., 2023; Dotov et al., 2021; Duman et al., 2024; Pfleiderer, 2010; Witek, 2017) (i.e., club listening). For a recent overview of previous groove literature, please see (Duman, 2023; Duman et al., 2024; Etani et al., 2024; Senn et al., 2023).

Importantly, recent work on individuals with anhedonia (Benson et al., 2024; Romkey et al., 2025) has shed light on the intricate nature of groove and pleasure, showing that musical anhedonics report lower overall pleasure and movement ratings. Using BMRQ, Benson et al. (2024) found that the sensorimotor dimension of musical reward was the only factor to predict movement ratings (but not pleasure), indicating that reward sensitivity is multidimensional and that pleasure and the urge to move are supported by overlapping yet distinct mechanisms. This may be because, while pleasure and movement in groove are often linked, they can depend on partly separate reward pathways: while pleasure is tied to dopaminergic mesolimbic circuits influenced by familiarity, prediction, and emotional appraisal; urge to move engages sensorimotor networks coupling auditory and motor areas and is reinforced by the intrinsic reward of bodily synchronisation. This suggests a shared predictive foundation but specialised mechanisms for hedonic valuation versus action preparation. In contrast, another study found no overall difference between anhedonics and controls in pleasure or movement ratings, with mediation analyses suggesting that in anhedonics, pleasure from groove may be sustained via the urge to move (Romkey et al., 2025). These findings highlight the need for further research into the complex, pleasurable nature of groove, looking beyond pleasure and movement alone to consider additional underlying mechanisms.

## The current work

Despite the extensive body of research on groove, studies often assess it primarily through ratings of pleasure and the desire to move, lacking granularity regarding other facets of the experience (for a detailed discussion, please see: Duman et al., 2024). However, given the various sources of pleasure discussed in the previous chapter, it stands to reason that the rewarding experience of groove is equally complex. Following the granular definition of groove proposed by Duman et al. (2024), the present work further develops a theoretical argument briefly introduced in Duman (2023): that the experiential dimensions of groove may be linked to distinct yet interlinked sources of pleasure arising during a groove experience. To better capture this multifaceted nature, the present work builds on broader theoretical and empirical perspectives from the musical pleasure literature. By integrating insights on reward mechanisms, predictive processing, peak emotional responses, embodied and sensorimotor engagement, and social bonding, this paper aims to develop a comprehensive framework that identifies and organizes the diverse sources of pleasure within the groove experience. Since groove may be experienced in different forms, such as solitary listening, collective dancing, or musical performance, the proposed sources are not assumed to be equally present or expressed in the same way across all cases. Rather, their relative contribution may vary depending on the context of the experience. Accordingly, four primary sources of pleasure will be outlined: pleasure derived from (1) perception of music, (2) experience of an immersive state, (3) movement, and (4) feeling socially connected. These operate at biological (cognitive and bodily), psychological, and social levels, respectively, reflecting the multidimensional character of groove.

## Perception of music on the cognitive level

“A mind is fundamentally an anticipator, an expectation-generator” (Dennett, 1996, p. 57). This becomes especially evident in the cognitive processing of music, where anticipation of future events shapes perception and experience, as listeners continuously form predictions about upcoming sounds based on learned structures and conventions. According to *theory of expectations* (Meyer, 1956), emotions in music arise from the dynamic interplay of tension and release: when listeners’ expectations are fulfilled, they experience resolution and satisfaction, whereas delays, suspensions, or violations of expectation generate tension, surprise, or heightened arousal. Building on this, Huron’s *ITPRA model* (Huron, 2006) distinguishes five stages (imagination, tension, prediction, reaction, and appraisal) that capture how listeners anticipate musical events, experience physiological arousal while waiting, respond emotionally when expectations are confirmed or violated, and later reflect on those experiences.

Complementarily, *dynamic attending theory* (DAT) (Jones and Boltz, 1989; Jones et al., 2002; Large and Jones, 1999) provides a mechanistic explanation of how temporal attention aligns with rhythmic patterns to support the formation and fulfilment of musical expectations. It proposes that attention is inherently rhythmic and can be entrained by external temporal patterns, such as music. In this view, attention oscillates in time with rhythmic structures, leading to heightened expectation and perceptual sensitivity at anticipated moments. Research has shown that perception and memory are most accurate for events occurring at expected points in time, suggesting that expectation heightens attention, which in turn facilitates perception and memory (Jones et al., 2002). While DAT provides a cognitive-attentional account of how listeners synchronize their focus with external rhythms, the *predictive coding framework* (Friston, 2010) offers a neurocomputational account of how the brain continuously generates predictions about sensory input to guide perception and action. It is described as a survival mechanism in which the brain continuously generates predictions about incoming sensory input and updates its internal models by minimizing the mismatch (prediction error) between expected and actual signals, thus maximizing the accuracy of future predictions (Vuust and Witek, 2014). From this standpoint, entrained attentional oscillations, as described by DAT, can be understood as a temporal mechanism through which the brain establishes priors, allocates resources efficiently, and reduces surprise in response to rhythmic auditory input (Koelsch et al., 2019).

Predictive coding is therefore directly linked to reward, since pleasure often emerges from the interplay between fulfilled and violated expectations, especially in the context of music. Successful predictions reduce uncertainty and stabilize internal models, while subtle deviations generate optimally weighted prediction errors that stimulate dopaminergic reward pathways (Vuust and Witek, 2014; Witek et al., 2017; Zatorre and Salimpoor, 2013; Berridge and Kringelbach, 2015; Kringelbach and Berridge, 2009). Neuroimaging evidence (Salimpoor et al., 2011, 2013, 2015; Zatorre and Salimpoor, 2013; Cheung et al., 2019) shows that this balance activates the nucleus accumbens, ventral striatum, and auditory cortices. These regions play complementary roles: the nucleus accumbens is central to reward valuation, the ventral striatum contributes to motivational processes that link reward anticipation with action, and the auditory cortices extract structural regularities and generate predictions about upcoming sounds. Their coordinated activity supports the view that musical enjoyment emerges at the sweet spot between predictability and surprise (Stupacher et al., 2022). Importantly, dopaminergic activity has been shown to peak both in anticipation of rewarding musical moments and during their occurrence, indicating that pleasure arises from the predictive buildup as well as from the peak experience itself (Salimpoor et al., 2011). During music perception, auditory stimulation acts as a ‘psychoactive trigger’ that recruits neurochemical systems (such as dopamine, opioids, and stress-related transmitters) to regulate arousal and reward, thereby linking sensory input to hedonic and homeostatic processes (Reybrouck and Van Dyck, 2024). In peak experiences, music may amplify these responses, evoking neurochemical cascades comparable to those observed in psychedelic or altered states, yet mediated through sensory and cognitive channels rather than direct pharmacological action (Reybrouck and Van Dyck, 2024). Recent work further supports this link by showing that individuals with substance use disorders, whose dopaminergic reward systems are altered, experienced stronger groove in response to high rhythmic and harmonic complexity than nonusers, suggesting that groove may be shaped by individual differences in reward thresholds and complexity processing (Stupacher et al., 2025).

Extending predictive coding to rhythmic engagement, groove studies show that motor and reward networks work closely together (i.e., Stupacher et al., 2013). This involves cortico-striatal circuits, along with prefrontal and parietal regions, which support both beat-based expectations and affective responses, with the basal ganglia playing a central role in generating the sensation of groove (Matthews et al., 2020). Within this framework, the role of syncopation in groove can be understood; moderate syncopation elicits the strongest experience because it preserves a salient metrical framework while introducing sufficient violations to generate prediction errors that are strongly weighted due to high metrical certainty. By contrast, low levels of syncopation minimize prediction errors, whereas high syncopation weakens metrical expectations and reduces their salience, so medium levels constitute the sweet spot for maximal reward (Vuust and Witek, 2014; Matthews et al., 2019; Stupacher et al., 2022). What is more, it has been suggested that moving to music helps reduce these prediction errors; this point led us to the movement aspect, which will be elaborated in the section on movement. Giving another account on this, Matthews et al. (2023) argue that the pleasurable urge to move to music (PLUMM) is supported by an intrinsic motivation for learning progress, to reduce prediction errors over time (see also: Berridge and Kringelbach, 2015; Kringelbach and Berridge, 2009). When listeners encounter rhythms that produce reducible prediction errors, these indicate opportunities for learning, evoking a pleasurable, curious state that recruits attention and memory. To formalize this idea, the authors propose a neuroscientific feedback model in which dopamine and norepinephrine mediate a loop connecting prediction-based learning, curiosity, and memory, thereby accounting for how PLUMM arises.

Complementing these accounts, empirical studies have demonstrated converging neural and physiological mechanisms underlying groove. Zalta et al. (2024) showed that medium syncopation elicited the strongest urge to move, reflecting an inverted U-shaped relationship between complexity and groove. Results revealed that, beyond beat tracking in auditory regions, cross-frequency coupling between delta and beta oscillations in the dorsal auditory-motor pathway, coordinated by the left sensorimotor cortex, was critical for linking temporal predictions with motor engagement. These findings align with a neurodynamic model, suggesting that oscillatory motor engagement during music listening reflects predictive timing shaped by interactions within the dorsal auditory pathway. Moreover, physiological evidence from pupillometry studies indicate that groove-related stimuli also modulate arousal via noradrenergic mechanisms. Bowling et al. (2019) reported stronger pupil dilations for high- versus low-groove music, while Spiech et al. (2022) observed weakened entrainment with increasing rhythmic complexity but sustained dilations peaking at medium complexity. These results suggest that groove recruits both attentional oscillations through predictive and neurochemical mechanisms to support the pleasurable aspect of groove experiences on the cognitive level.

## Immersion on the psychological level

Musical involvement has been reported as one of the most frequently mentioned peak experiences (Lowis, 2002). One notable quality of such experiences is the sense of “getting lost in the present (…) detachment from time and place” (Maslow, 1971, p. 64). Moreover, absorption in music is linked with musical enjoyment (Rhodes et al., 1988; Sandstrom and Russo, 2013). Therefore, as outlined in the previous section, while perception of music on a cognitive level generates expectations and activates relevant brain regions, it may also induce an immersive, flow-like, and rewarding state on a psychological level. These expectations generated through the musical structure invite the listener to engage with the music (Duman et al., 2024). Similarly, Roholt (2014) describes how timing nuances in the music give rise to a felt dimension; experienced as ‘leaning’, ‘pushes’, and ‘pulls’, that deepens the listener’s engagement and contributes to the immersive quality of the groove. Danielsen (2006) elaborates on by describing how this invitation arises from the tension produced between the main beat and counter rhythm. The listener then resolves this tension either through bodily movement or mental synchronization, which will be elaborated on in the next section.

Previous literature has pointed out that the term immersion has been used across disciplines (such as virtual reality and interactive multimedia) and often inconsistently, referring interchangeably to presence or engagement (Nilsson et al., 2016). Other terms closely related to immersion are *absorption* (extreme involvement) and *presence* (being there) (Tellegen and Atkinson, 1974; Wild et al., 1995; Wycisk et al., 2022). Similarly, the state of *flow* is described as a high level of involvement and full concentration, resulting from a balance between the skills of the engager and the challenge of the task (Csikszentmihalyi and Csikzentmihaly, 1990). The distinction between a flow state and an immersed state is based on the type of activity being engaged in; specifically, whether it involves passive or active participation (Agrawal et al., 2020). Although immersion can be a challenging concept to define precisely, a metaphor may offer a more intuitive understanding of the term:

*“Immersion is a metaphorical term derived from the physical experience of being submerged in water. We seek the same feeling from a psychologically immersive experience that we do from a plunge in the ocean or swimming pool: the sensation of being surrounded by a completely other reality, as different as water is from air, that takes over all of our attention, our whole perceptual apparatus.”* (Murray, 1997, p. 98, as cited in Nilsson et al., 2016).

While providing detailed distinctions among these closely related concepts is beyond the scope of this paper (and will be tackled in another study: Duman et al., 2025), the term immersion is used here in line with the metaphor of being submerged in or surrounded by water, akin to the experience of cognitive, bodily, and emotional involvement or connection with the music (Duman et al., 2024; Nilsson et al., 2016; Wycisk et al., 2022).

The immersive aspect of the groove experience has been overlooked and under investigated in previous groove literature (Duman et al., 2024). Although research in this area remains limited, both theoretical and empirical interest is growing. Conceptually, groove has been described as emerging in the immediacy of performance, making it difficult to fully grasp; as soon as one becomes self-aware or attempts to analyse the experience, they are no longer entirely immersed in it (Câmara and Danielsen, 2018). Scarcity of research on the immersive aspect of groove might be also due to this fragility of the experience (for a further explanation, see Duman et al., 2024). Nevertheless, empirical studies show the link between groove and immersive states. A qualitative investigation identified immersion as a core experiential component of groove, reflected in participants’ descriptions such as “something that hooks me,” “feeling different from the present,” and “when you understand and get into the flow of the song” (Duman et al., 2024, p. 106). Complementarily, in an interview study with musicians, Bechtold et al. (2023) reported that immersion in groove is deeply intertwined with participation and social connection. Immersion was described as both a cognitive and bodily state in which listeners feel captivated or ‘caught’ by the music. A finger-tapping study (Stupacher, 2019) demonstrated that sensorimotor synchronization accuracy and stability were linked to the fluency dimension of flow, particularly with medium and high rhythmic complex stimuli, highlighting the role of perception-action coupling during the flow experience. In agreement with the concept of flow, moderate amount of rhythmic complexity is deemed to introduce a level of challenge that promotes a focused, flow-like or immersive mental state, an experience that is intrinsically rewarding (Stupacher et al., 2022; Vuust and Witek, 2014; Witek et al., 2014).

Further evidence from the broader musical pleasure literature may support the proposed relationship between immersive states and enjoyment during groove experiences. According to Brattico and Pearce, 2013, an aesthetic experience of music is formed when one immerses in music perceptually, cognitively and affectively; resulting in three outcomes: (1) emotion recognition and induction, (2) aesthetic judgement, and (3) liking and preference. While frontotemporal areas get involved in musical processing; limbic, paralimbic and reward circuits are activated during emotional responses and pleasurable experiences to music on the psychological level. What is more, at higher intensities, immersive musical states may resemble altered states of consciousness, involving time dilation, timelessness, or distortions in spatial perception, similar to meditation or drug-induced states (Schäfer et al., 2013a). Such experiences highlight the embodied nature of music listening, as bodily engagement shapes subjective representations of time and presence. According to Schäfer et al.’s model, music influences time perception by modulating temporal interval processing and the sense of ‘nowness’. Deep absorption may diminish perceived gaps between moments, producing a more continuous flow of time. This process is further modulated by neurotransmitters (e.g., dopamine, serotonin, norepinephrine, acetylcholine), mood, context, and individual differences (Wittmann et al., 2007a, 2007b; Wackermann et al., 2008; Arstila, 2012; Meck, 1996). For instance, individuals with a higher tendency for being absorbed in music are also more likely to experience musical reward (Cardona et al., 2022). Regarding context, Vroegh et al. (2021) found that being absorbed in popular music can enhance manual reaction times during a visual task, suggesting that musical absorption may induce a flow-like state that improves motor performance, whereas mind wandering tends to slow responses.

Research from the field of psychology complements this finding people are happiest when their minds are in the present moment, not wandering (Killingsworth and Gilbert, 2010), which has been reported to promote lowering distress (Kiken et al., 2015). Musical immersion is also a psychologically safe experience, because it allows simultaneous absorption and dissociation (Garrido and Schubert, 2013). Saarikallio (2019) argues that in music one can feel deep experiential surrender to the embodied excitement, yet keep one’s cool by stating “it was just a song.” One can operate at an optimal, tolerable window of experiential depth, fluently negotiating between immersion and self-detachment, and feeling agency of the internal experience. Thus, immersion may contribute to groove-related pleasure by allowing listeners to become deeply absorbed in the musical moment, while still retaining a sense of safety, self-detachment, and agency.

## Movement on the behavioral level

Empirical studies on music-induced movement show that musical elements such as meter, rhythm, volume, and low-frequency sounds are reflected in bodily motion, with rhythmic structure encoded across multiple metric levels and shaping movement patterns (Burger et al., 2013, 2018; Toiviainen et al., 2010; Toiviainen and Carlson, 2022; Mårup et al., 2022; Stupacher et al., 2016; Hove et al., 2020; Cameron et al., 2022). For instance, bass drum dynamics have been shown to energize whole-body movements and strengthen tempo entrainment in social contexts (Van Dyck et al., 2013; McNeill, 1997; McCown et al., 1997). This effect was linked to the vestibular system: the sacculus is highly sensitive to low-frequency vibrations, and its activation has been associated not only with bodily responses but also with the emergence of musical pleasure (Todd et al., 2008). These findings exemplify that musical features are embodied in movement, emphasizing the close coupling of perception, action, and reward in musical experience.

Rhythm perception is inherently multimodal, engaging not only the auditory system but also other modalities, especially the bodily movement playing a particularly central role. Fitch (2016) for example, argues that rhythm and groove cannot be understood apart from their embodied roots in dance, emphasizing how metric structure is tied to movement, as in African *agbekor* drumming where motion is vital for feeling the pulse. He reframes syncopation not as an abstract device but as an acoustic reflection of off-beat dance gestures, highlighting the inseparability of sound and movement. An empirical study reported that bodily movements, such as bouncing on every second versus third beat of an ambiguous rhythm, shape whether it is heard in duple (march) or triple (waltz) meter, even from infancy onwards (Phillips-Silver and Trainor, 2005, 2007). Extending this, Cheng et al. (2022) showed that hierarchical rhythm perception arises from dynamic auditory–motor interactions even without movement: during imagined metres, motor regions synchronized at the beat level and drove information to auditory areas. Theoretically, these findings are supported by the *action simulation for auditory prediction* (ASAP) hypothesis (Patel and Iversen, 2014), arguing that beat perception is a complex neural function that relies on precise temporal interactions between auditory and motor regions, even when no actual movement occurs.^2^ Periodic movements are internally simulated within motor planning regions, generating signals that aid the auditory system in predicting the timing of upcoming beats. (For a recent review on neuroscience of dance, see: Vander Elst et al., 2023.) This top-down influence is supported by *embodied cognition* theories too (Leman, 2007; Maes et al., 2014; Maes, 2016), positioning the motor system as central to constructing hierarchical timing. Rhythm is thus not only ‘heard’ but also ‘felt’ through covert motor simulation, grounding predictive timing structures in sensorimotor processes.

Linking the ASAP framework with predictive coding perspectives discussed earlier, the body can be understood as an active aid to auditory prediction, optimizing the estimation of future events. This perspective leads directly to the theory of *active inference*, which extends predictive coding by reframing perception as an active, top-down process rather than a passive one. Under this account, cognition (prediction, planning, action, learning) and perception form an inseparable hierarchy of dynamics aimed at reducing prediction error (Koelsch et al., 2019). Friston (2010) and Friston et al. (2017) proposed that perception and action are unified under the free-energy principle, which posits that biological agents minimize ‘free energy’ (a bound on surprise) by continuously updating internal models to match sensory input while actively sampling the environment to confirm predictions. Within this framework, motor control arises from prior expectations about proprioceptive states, realized through reflexes and intentional movements rather than explicit optimal control computations, while neuronal dynamics more broadly can be explained as the continuous minimization of variational free energy, providing a unifying account of neural phenomena and adaptive behaviour. Supporting this view, Brown et al. (2011) suggested that action planning can be understood as an attentional process directed toward sensorimotor signals, with such biases operating early in the motor hierarchy in line with active inference accounts of proprioception. This implies that movement is guided by predictions of expected sensory and motor feedback rather than by the computation of explicit motor commands.

Situating the evolutionary theory, ecological psychology, and embodied phenomenology within the framework of active inference, Linson et al. (2018) argue that perception-action systems minimize uncertainty to enhance survival. From this view, organisms directly perceive affordances, and cognition is grounded in lived bodily engagement with the world, linking first-person experience to predictive, action-oriented dynamics. Building on, Hesp et al. (2021) introduces the notion of *affective inference*, suggesting that emotional valence reflects how confident an agent is in its ability to act effectively, with changes in this confidence (termed as affective charge) shaping action choices and linking affect directly to predictive, embodied processes. This implies that valence functions as a dynamic signal of model confidence: positive valence indicates high confidence, negative valence reflects diminished confidence, and fluctuations in affective charge track how well predictions are confirmed. Rather than being a by-product, emotion actively guides action selection by tuning the weight we place on predictions.

Appropriating this concept to music, it can be viewed as a unique source of reward, continuously satisfying our need to resolve uncertainty. Within a predictive coding framework, this is explained through precision weighting: listeners not only predict upcoming events but also adjust their confidence in those predictions, making active listening central to music’s rewarding nature (Koelsch et al., 2019). Groove in particular has been discussed to engage the brain’s predictive mechanisms, as features such as syncopation, polyrhythm, and rhythmic complexity evoke both pleasure and the urge for sensorimotor synchronization; arising from a balance of predictability and surprise, producing an inverted U-shaped relationship between rhythmic complexity and pleasure (Vuust and Witek, 2014; Stupacher et al., 2022). Conceptually, Witek argues that syncopation in music affords the body to fill in these gaps (Witek, 2017). This notion will be elaborated in the next section. Testing active inference empirically, an EEG tapping study (Ishida and Nittono, 2025) found that participants listening to syncopated rhythms showed larger mismatch negativity (MMN), higher subjective pleasure and energy ratings when tapping compared to a no-tapping condition, indicating that bodily engagement (tapping) enhances beat perception, modulates rhythmic prediction error and emotional state. These suggest that the pleasure of groove may arise from heightened confidence in predicting and synchronizing with rhythm, while a disruption of groove experience could reflect diminishing confidence in these predictive models. Crucially, this confidence also shapes the type and extent of bodily movements we engage in while listening, which in turn feed back into the affective state, creating a dynamic loop between movement, prediction, and pleasure. Finally, in addition to findings from the musical domain, evidence from sports and exercise literature shows that bodily movements, such as physical exercise and dance activate multiple neurochemical systems (such as dopaminergic, endorphinergic, and endocannabinoid pathways) that contribute to pleasurable and rewarding affective states, supporting stress relief and the mild euphoria often associated with exercise (Dietrich and McDaniel, 2004; Matei et al., 2023; Siebers et al., 2023).

## Connection on the social level

Humans have long been recognized as inherently social beings, as Aristotle famously termed “zoon politikon.” This description links our biological nature with psychological flourishing and highlights the necessity of community in this relationship. In other words, human experiences cannot be discussed in isolation, separating the individual from its social settings. In line with this, social psychology research shows that people unconsciously adapt to others through mechanisms such as the *chameleon effect* (described as nonconscious mimicry of postures, expressions, and movements) which enhances liking, smoother interactions and social bonding (Chartrand and Bargh, 1999). Bamford (2022) suggests that one mechanism underlying synchrony-induced social bonding is processing fluency, whereby coordinated movements reduce cognitive load, enhances perceived ease, and cultivates a sense of connection. This idea resonates with theoretical accounts that integrate predictive processing and active inference in social contexts (Ramstead et al., 2016; Albarracin et al., 2024), beyond the individual level discussed above. From this perspective, social connection emerges through shared predictive processes: cultural practices and regimes of attention align expectations and focus (Ramstead et al., 2016), while shared forward-looking predictions, or protentions, ground collective goals and group intentionality (Albarracin et al., 2024).

With respect to experience of groove and social factors, despite the direct research evidence being rather scarce, several studies highlighted the influence of social context on experiencing groove. In a qualitative study, among its other components, participants described the groove experience with its social aspect: “Bonding you to the people who are also grooving at the same time.” (Duman et al., 2024, p. 107). In another qualitative study with musicians, Bechtold et al. (2023) reported groove as a collective, participatory, and immersive experience that emerges through interaction between musicians and audiences. Rather than an individual achievement, groove is understood as a shared effort that builds trust and community, often expressed through participatory acts like clapping or dancing, as well as immersive states of being captivated by the music.

On a theoretical level, Witek (2019) uses raves in club settings to illustrate how people come together and experience the ‘synchronising force’ of music and atmosphere, accompanied by heightened ecstasy, timelessness (a sense of eternal present or now-ness), and the dissolution of self into a collective body, feeling one. Witek (2017) explains this in terms of musical features such as volume, the tactile impact of bass, and especially the rhythmic gaps in syncopated music, which invite the body to move in ways that ‘fill in’ the missing beats, thereby making dancers active participants in the musical structure (see also Stupacher et al., 2022). By enacting the beat physically (or virtually through embodied attention when listening), the boundaries between mind, body, and music dissolve. This demonstrates how individual music perception at cognitive, psychological and bodily levels translate into synchronous movement across multiple individuals in a group setting. This collective movement signals trust among the participants, elevating the experience (Witek, 2019).

While Witek emphasizes the sensory and musical drivers that enable such collective experiences, the concept of *joint action* provides a complementary cognitive framework for understanding how these processes unfold. Joint action refers to the ability of humans to coordinate their behaviour to achieve shared goals, supported by mechanisms such as joint attention (sharing perceptual focus), perception-action coupling (representing and predicting others’ goals), shared task representations (anticipating actions from environmental cues), and the alignment of “what” and “when” in action planning (Sebanz et al., 2006). These mechanisms show how smooth coordination depends on integrating self and other actions, often prioritizing collective outcomes over individual ones. In this way, joint action highlights the cognitive and neural foundations that underlie the synchrony and trust observed in musical and dance settings.

Extending this view, it has been discussed that moderately syncopated rhythms are especially effective in balancing group synchrony and individual creativity: while strong beats enable shared metrical predictions that support joint action, off-beat notes and pauses open space for variation and improvisation (Stupacher et al., 2022). This balance not only provides a temporal framework for coordinated group movement but also creates room for personal expression, therefore enhances shared emotional experiences. This view can also be interpreted through self-determination theory (Deci and Ryan, 2012), which holds that satisfying the basic psychological needs of autonomy, competence, and relatedness cultivates intrinsic motivation and supports well-being. Moderately syncopated rhythms, by providing both a common temporal framework and room for personal expression, help fulfil these needs and transform coordinated movement into a pleasurable and socially rewarding groove experience.

Building on this theoretical stand, empirical studies further demonstrated that shared activities such as music listening and making, dance, coordinated movement, and shared emotions have been shown to play a central role in promoting social identity, prosocial behaviour, bonding, and connection (Arewasikporn et al., 2019; Lee et al., 2020; Marsh et al., 2009; Savage et al., 2021; Solberg and Jensenius, 2017a; Stupacher et al., 2016; Stupacher et al., 2022). People tend to feel greater affinity toward others who share similar musical preferences (Boer et al., 2011). Kowalewski et al. (2020) demonstrated that perceived groove is also shaped by affinity toward the performing musician. They showed that moderately syncopated, high-groove drum breaks were rated as more pleasurable and movement-inducing when attributed to a likable versus an unlikable musician, whereas this effect disappeared with highly syncopated, low-groove rhythms. This finding indicates that groove and musical pleasure are not determined by rhythm alone but are influenced by the listener’s motivation to affiliate with the performer (this effect emerges only when the music is sufficiently groovy to begin with). In other words, these studies demonstrate that groove experiences are shaped not only by musical structure but also by social dynamics. Motion capture research highlights this interplay too: in EDM, structural elements such as breakdowns, build-ups, and drops elicited stronger group synchronization, and participants reported that the presence of others influenced their own experience (Solberg and Jensenius, 2017b). Similarly, Dotov et al. (2021) found that groove ratings, movement energy, and interpersonal connection increased when participants could access social cues in the eyes-open compared to the eyes-closed condition. Using a silent disco paradigm, Bamford et al. (2023) showed that dyads moving in synchrony reported stronger partner interaction and engaged in more mutual gaze than when moving out of synchrony, suggesting that sharing time, rather than merely space, is key to the social effects of joint dancing.

Movement, dance, and exercise result in the release of hormones such as endorphins (and interact with oxytocin) that are closely linked with affective states. For example, Dunbar et al. (2012) showed that engaging in active musical performance (e.g., singing, dancing, drumming) elevates pain-threshold (used as a proxy for endorphin release) and increases positive affect, whereas passive listening or low-energy musical activity did not do so. Tarr et al. (2014) and colleagues further argue that rhythmic, synchronized movement not only promotes endorphin release but also strengthens social bonds through a “self–other merging” mechanism. In their framework, synchronized movement allows individuals’ motor systems to co-activate, blurring the boundary between self and other, making it particularly rewarding both emotionally and socially. These bonding experiences can further lead to prosocial actions, such as in Kirschner and Tomasello’s (2010) work with 4-year-olds, who showed increased prosocial actions towards their peers after shared, synchronised movement to music. Overall, these works demonstrate that individual experiences of music in group settings can translate into increased synchrony, affiliation, and social bonding, which signal trust, safety, and cohesion. From an evolutionary perspective, such coordinated group behaviours enhance survival by strengthening social ties and are therefore intrinsically rewarding (Dunbar, 2012; Savage et al., 2021; Tarr et al., 2014).

## Summary: the four pleasures of groove

**Figure 1:**
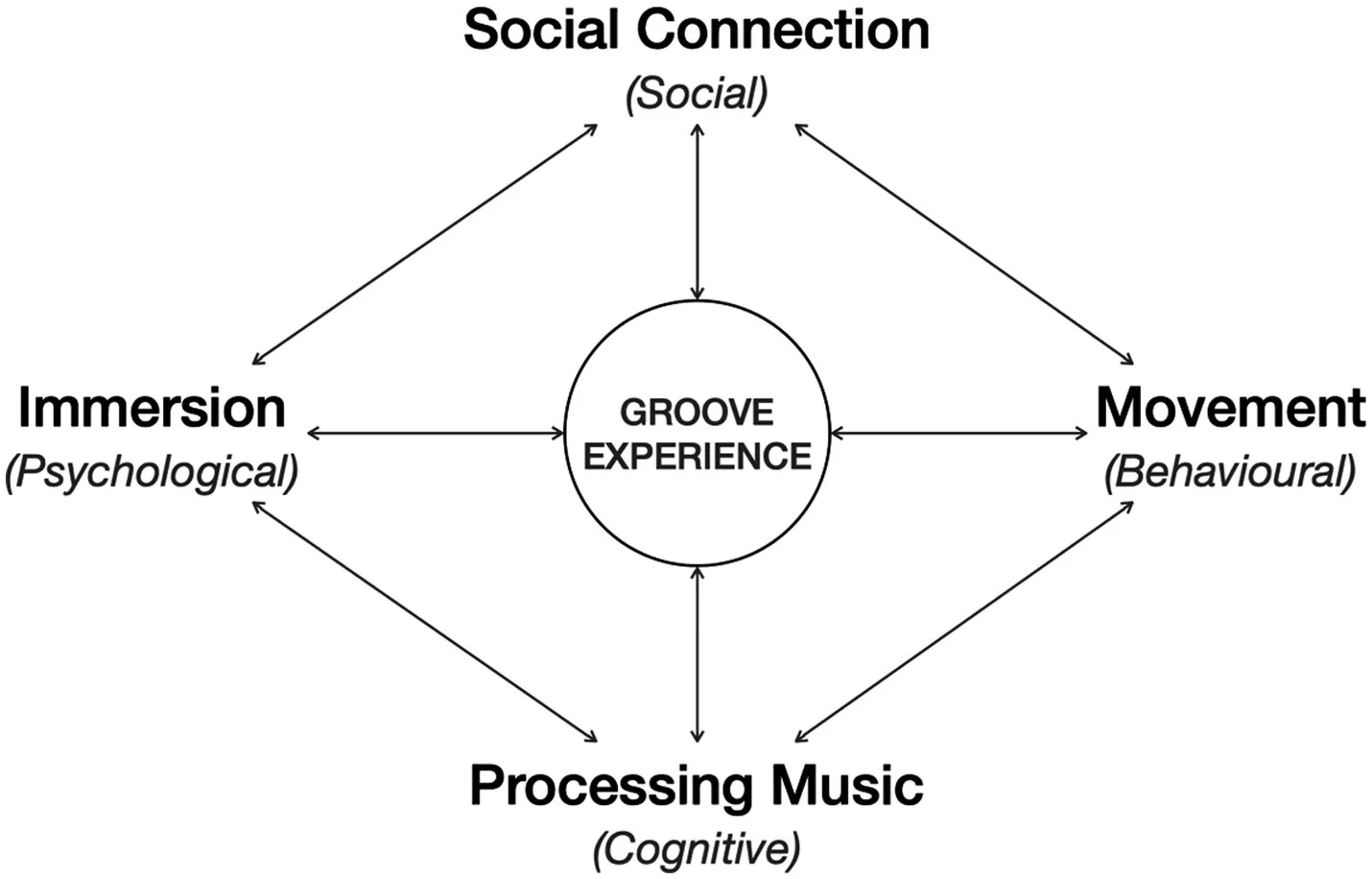
The four sources of pleasure associated with the experience of groove. Pleasure from processing of music arises when predictive mechanisms balance expectation and surprise, engaging reward pathways that heighten attention and invite further involvement. This engagement on the cognitive level promotes an immersive state on the psychological level, where focused attention deepens the experience and enhances its rewarding qualities. On the behavioural level, bodily synchronization reduces prediction errors and provides intrinsic sensorimotor pleasure. On the social level, one might experience heightened trust, affiliation, and bonding through synchronised movements. These four sources of pleasure are interconnected, feeding into one another, and highlight the multidimensional nature of groove that encompasses cognitive, psychological, bodily, and social pleasures.

Combining theoretical accounts and empirical findings across multiple fields, we propose four interrelated sources of pleasure, illustrated in Figure 1, that operate at different levels during a groove experience. These sources are distinguished by the primary level at which pleasure is experienced and expressed: cognitive (arising from the processing of musical structure and prediction), psychological (linked to an immersive state), behavioral (emerging from the urge to move and embodied interaction with rhythm), and social (stemming from coordination, synchrony, and shared affect with others). These are further supported by underlying physiological mechanisms, with neurochemical systems such as dopamine, norepinephrine, serotonin, and acetylcholine modulating attention, arousal, temporal processing, and reward, thereby anchoring the pleasures of groove in embodied brain–body dynamics.

## Discussion

The current paper presents a conceptualization of groove-induced pleasure as a multidimensional experience. The four levels presented help to conceptually separate the constituents of pleasure in groove, advancing knowledge of the granularity of this experience, while the model simultaneously highlights their mutual and integrated presence. The proposed model renews the conceptualization of groove-induced pleasure, expanding the dichotomic view of pleasure and urge to move into a more granular and holistic approach. Integration of cognitive, embodied, psychological, and social levels embraces the multilayered character of human experience. It aligns with views of human experience of presence as a layered system of bio-cultural mechanisms such as bodily sensorimotor coupling, situational awareness, and narrative and reflective meaning-making (Riva et al., 2004).

Although this approach provides a comprehensive account, it also entails certain challenges. The constitution of the pleasure experience may be more strongly driven by any of the proposed components, varying across individuals but also across situations and momentary attentional processes (Biocca, 2003; Waterworth and Waterworth, 2001). The proposed model serves as a conceptual framework, encouraging future research to refine and validate it by examining the mutual presence and relative contributions of its components across diverse contexts and populations. Potential next steps for investigation include the following hypotheses for future studies to test:

1. Active versus passive engagement: Future research should examine the extent to which groove requires bodily involvement (e.g., movement, synchronization) versus whether it can be experienced passively through covert forms of multisensory engagement, and how the intensity of pleasure varies with these modes of participation.
2. Dimensions of immersion: Future research should explore how different dimensions of immersion shape groove, which may be experienced as bodily, cognitive, emotional, or social depending on context. Insights from presence research can inform this work: theories of presence describe mechanisms such as shifts between self, other, and environment (Biocca, 2003); attention directed outward, inward, or toward heightened awareness (Waterworth and Waterworth, 2001); layered forms of sensorimotor, situational, and reflective engagement (Riva et al., 2004); and the construction of immersive and plausible collective experiences (Slater, 2009). Systematic investigation is needed to clarify how these dimensions interact and whether groove manifests primarily as self-immersion, synchrony with others, or absorption in the musical environment (Nilsson et al., 2016).
3. Social context: Future research should investigate whether the experience of social connection in groove depends on being physically surrounded by others, or whether solitary engagement with groove-related music can similarly evoke a sense of bonding, given music’s inherently social nature (Boer and Fischer, 2012; Schäfer et al., 2013b).

Despite the open questions, offering a more granular view on groove-induced pleasure, the current model presents itself as a potentially fruitful framework for investigating and explaining individual and temporal differences in the groove experience. Considering the various types of pleasure a listener may get from experiencing groove might help us better understand this complex phenomenon. A granular conceptualization of pleasure also encourages detailed, granular measurement and potentially requires integration of paradigms that range from physiological measures to motion capture and subjective reports. A granular model of groove-induced pleasure also allows comparative research on the particular components of pleasure across activities, starting from comparisons of listening to different types of music to comparing listening and playing, and expanding to other activities beyond music engagement.

Beyond these insights, since humans are primarily hedonistic and as presented in this paper that groove can induce pleasure on multiple levels, future work should also consider pleasure itself as a key outcome measure, rather than focusing primarily on movement-related responses.^3^ While movement has often been treated as the main dependent variable in groove research, predicting and explaining pleasure directly may provide a more comprehensive understanding of the phenomenon.

Integrating cognitive processing, experiential immersion, bodily movement, and social aspects into how we conceptualize groove-related pleasure also has important implications for practice. Groove-induced pleasure can be approached as a multilayered and granular experience. When a music educator wishes to nurture the joy and pleasure of playing in their students, they can simultaneously consider setting appropriate levels of cognitive challenge, encourage experiential immersion, allow and facilitate movement and bodily awareness while playing, and take advantage of creating a space for shared action. Yet, while it is useful for practitioners to identify these different layers, they specifically benefit from the simultaneous presence of them in a groove experience. Groove-induced pleasure is inherently multidimensional, making it a holistic and easily accessible source of enjoyment and motivation. This quality may be particularly valuable for promoting bonding in school settings or encouraging emotional involvement in therapeutic contexts through bodily empowerment, as highlighted in discussions of the role of pleasure in movement and dance therapy (Lowen, 2024). In general, a granular approach to pleasure may offer cost effective opportunities for a more elaborate identification and measurement of impact-mechanisms in health interventions. Primary prevention actions that aim to reduce common risk factors such as excessive stress, powerlessness, low social competence, diminished self-confidence, and weak support networks (Albee, 1982a, 1982b) would benefit from a more evidence-based assessment of the underlying impact mechanisms.

## Data Availability

The original contributions presented in the study are included in the article/supplementary material, further inquiries can be directed to the corresponding author.

## Footnotes

1 In this article, the word pleasure is used as an umbrella term to pleasure, happiness and reward. Pleasure, happiness, and reward, while interconnected, denote distinct concepts in psychology and neuroscience. Pleasure is a transient feeling of enjoyment or satisfaction, often linked to sensory experiences. Happiness refers to a more enduring state of well-being or contentment, encompassing a broader evaluation of life. Reward, on the other hand, involves a neural process that reinforces behaviors beneficial for survival and reproduction, often triggering pleasure and potentially contributing to happiness. Each operates at different levels of human experience, from immediate sensations to long-term life satisfaction. This broad understanding of pleasure is therefore used to frame groove as a rewarding musical experience that may operate across several levels of human experience. Groove may involve immediate enjoyment, reward-related motivation to move and participate, and momentary feelings of happiness or well-being.

2 Due to this, as framed initially in Duman et al., 2024, the described movement-related pleasure includes both the felt desire or sense of movement and actual bodily actions such as dancing, nodding, swaying, singing, or jamming.

3 This idea was first introduced by Prof. Petr Janata in the Groove Workshop, 2023, online.
